# Comparative In Vitro Osteogenic Capacities of Bone Marrow- and Periosteal-Derived Progenitor Cells

**DOI:** 10.3390/biology14101354

**Published:** 2025-10-02

**Authors:** Kalyn Herzog, Julie Nguyen-Edquilang, Matthew Stewart

**Affiliations:** 1 Veterinary Clinical Medicine, University of Illinois, Urbana, IL 61802, USA; kherzog@clcillinois.edu (K.H.); jknguyn2@illinois.edu (J.N.-E.); 2 Biological & Health Sciences, College of Lake County, Grayslake, IL 60030, USA

**Keywords:** osteogenesis, periosteum, bone marrow-derived stem cell, osteoprogenitor

## Abstract

**Simple Summary:**

Cartilage- and bone-forming cells from the periosteum are responsible for increasing bone width during skeletal growth, and fracture repair is also primarily orchestrated by these cells. These activities make periosteal cells excellent candidates for cell-based strategies to improve fracture outcomes. With this in mind, we compared the in vitro osteogenic (bone-forming) capacity of adult equine periosteal cells with that of bone marrow-derived stem cells. Against expectations, periosteum-derived cells exhibited little or no osteogenic activity while bone marrow-derived cells demonstrated robust osteogenesis. Further, the administration of the osteo-stimulatory growth factor BMP-2 was not sufficient to restore the in vitro bone-forming capacity of periosteal cells. Collectively, these outcomes demonstrate that isolation of periosteal cells from native tissue and/or in in vitro expansion of these cells have profound negative effects on their osteogenic capacity, an effect not seen in bone marrow-derived progenitors. Until appropriate phenotype-sparing isolation protocols are developed for periosteal cell isolation, bone marrow-derived cells should be preferred for cell-based strategies to facilitate bone repair.

**Abstract:**

Fracture repair complications occur in 5–10% of cases, despite bone’s regenerative capacity. Bone marrow-derived (BM) stem cells have been extensively investigated for orthopedic applications but, given the critical role that periosteum plays in fracture repair, periosteal-derived (PO) cells offer a promising alternative cell source. This study compared the in vitro osteogenic capacities of equine BM and PO cells. Passage 3 cells from each source were maintained in osteogenic medium for up to 10 days. Osteogenesis was assessed by Runx2, Osterix, and alkaline phosphatase (ALP) mRNA up-regulation, induction of ALP activity, and matrix mineralization. Comparisons were made by paired *t* tests, repeated measures one-way or two-way ANOVAs, as indicated. BM cells proved superior to PO cells in osteogenesis assays. BM cells significantly up-regulated Runx2, Osterix, and ALP mRNAs, ALP activity, and secreted a mineralized matrix by day 10. PO cells did not. BMP-2 expression increased significantly in BM cells in osteogenic medium, whereas BMP-2 expression in PO cells was unchanged. Exogenous BMP-2 did not restore osteogenesis in periosteal cells, indicating that ex vivo expansion affects periosteal osteogenic capacity beyond BMP-2 downregulation. Clinical applications of PO cells will require the identification and exogenous provision of requisite stimulatory factors and substrates.

## 1. Introduction

Osteogenesis is the process by which cells synthesize mineralized tissue and is one of the core phenotypic activities that define mesenchymal stem cell populations [1]. Prior to the onset of puberty, osteoprogenitors within endosteal metaphyses are active in converting the hypertrophic zone of growth plate cartilage into primary bone, resulting in bone lengthening. During skeletal growth, osteoprogenitors in periosteum are responsible for appositional bone formation to increase bone width. Periosteal osteoprogenitors are also responsible for intramembranous ossification of non-appendicular ‘flat’ bones [2]. After puberty, mesenchymal stem cells within the bone marrow compartment (BM) remain active in trabecular bone homeostasis while periosteal cells (PO) regulate external cortical bone thickness. Of particular clinical relevance, periosteal osteoprogenitors are the major cell population responsible for callus development and endochondral ossification during fracture repair [3,4,5]. Periosteal cells generate both the cartilaginous and osseous callus tissue within the external callus, reflecting their bi-lineage phenotypic capacity [5,6].

A recent study encompassing medical records from 204 countries over two decades calculated that the current fracture-related caseload burden exceeds 170 million, with expectations that this figure will increase as age-associated osteoporosis becomes a more contributory factor in an aging global population [7]. Although bone is one of the few tissues with authentic regenerative capability, an estimated 5–10% of fractures fail to heal within a clinically acceptable time frame [8,9]. With open long bone fractures, the complication rate approaches 20% [10]. In veterinary practice, fracture repair is also a major challenge, particularly in high-energy trauma cases consequent to vehicle accidents and fractures in equine athletes [11,12]. This has prompted a great deal of research focused on strategies to support and accelerate bone repair, including orthopedic hardware development, grafting, tissue engineering, and cell-based interventions. Although restoration of skeletal function is the primary goal of fracture management, there has been a growing awareness of the importance of periosteum and other soft tissue contributions to fracture repair and the need to protect peri-fracture soft tissues during fracture stabilization to optimize biological healing [13,14,15].

There is an extensive body of literature addressing the use of BM cells to support bone repair (see references [16,17] for recent authoritative reviews). However, in light of the central role played by periosteum in fracture healing, there is growing interest in and advocacy for utilizing PO progenitors to augment bone repair [18,19,20,21]. Self-evidently, for any cell-based strategy that requires ex vivo selection and/or expansion of specific cell populations, maintenance of osteogenic capacity prior to re-implantation is critical. We approached this issue by comparing the in vitro osteogenic capacities of donor-matched BM- and PO-derived cell populations isolated from adult horses, using the standard in vitro protocol used to characterize mesenchymal stem cell osteogenic competency. The study addressed the hypothesis that bone marrow-derived and periosteum-derived progenitor cells exhibit equivalent in vitro osteogenic capacities. Outcome measures focused on up-regulation of the lineage-determining osteogenic transcription factors, Runx2 and Osterix, induction of alkaline phosphatase (ALP) mRNA levels and enzymatic activity, and production of matrix mineralization [22,23].

## 2. Materials and Methods

### 2.1. Bone Marrow Aspirate and Periosteum Collection

All procedures were approved by the University of Illinois Institutional Animal Care and Use Committee. Cells were collected from five healthy adult horses aged between 2 and 10 years of age that were donated to our facility for research purposes. The horses were fasted overnight prior to sample collections. These horses were sedated with 1.0 mg xylazine/kg. Anesthesia was induced with a combination of 2.2 mg ketamine/kg and 0.1 mg midazolam/kg, administered intravenously through a jugular catheter. Horses were placed in right lateral recumbency, and oro-tracheal intubation was performed to allow for mechanical ventilation and general anesthesia maintenance with isoflurane delivered in oxygen.

Bone marrow aspirates were collected from the sternebrae [24]. The skin of the ventral sternum region was clipped and aseptically cleaned with chlorhexidine and 70% ethanol scrubs. A small stab incision was made using a #11 scalpel blade directly over the 5th sterebrae, just caudal to the tuber olecranon. A 12-gauge Jamshidi needle was introduced into the stab incision and advanced approximately 1–2 cm through the ventral cortical bone and into the medullary cavity of the sternebra. A 12 mL syringe preloaded with 5000 IU of heparin was connected to the Jamshidi needle and used to slowly aspirate 5–10 mL of bone marrow. The syringe was inverted several times, and then the bone marrow aspirate was transferred aseptically to a 50 mL sterile conical tube.

Periosteum was collected from the craniomedial aspect of the right tibia. After clipping and disinfection of the site, a cranially oriented curvilinear skin incision was made the length of the tibial shaft. The skin was reflected caudally to expose the tibial surface between the cranial tibialis muscle belly cranially and the medial gastrocnemius and digital flexor muscle bellies caudally. The subcutaneous and loose fascial tissue over the tibial shaft was excised until the tightly adherent periosteal surface was accessed. Periosteum was lifted from the cortical bone surface using a periosteal elevator and transferred to Dulbecco’s Modified Eagle’s Medium (DMEM; Corning Life Sciences, Kenebunk, ME, USA) supplemented with 10% fetal bovine serum (FBS; GemCell FBS, GeminiBio, West Sacramento, CA, USA), 2% penicillin/streptomycin (Pen/Strep; Gibco Thermo Fisher Scientific, Waltham, MA, USA) and 2.5 μg of amphotericin B (Gibco)/mL, on ice.

### 2.2. Sample Processing

Bone marrow aspirates were mixed and suspended in 30 mL of phosphate-buffered saline (PBS) containing 1% Pen/Strep, then centrifuged at 300× *g* for 15 min (Sorvall ST 16R Centrifuge, Thermo Fisher Scientific). The cell pellet was resuspended in growth media (DMEM, 10% FBS, 1% Pen/Strep) and an aliquot was used to count nucleated cell numbers from each collection. Resuspended cells were transferred to two T-75 cm^2^ cell culture flasks (Corning) to a volume of 15–20 mL per flask, at a nominal nucleated cell seeding density of 1 × 10^5^ cells/cm^2^. Flasks were maintained in an incubator at 37 °C in 5% CO_2_. For the first week, about half of the supernatant was removed and replaced with 10 mL fresh growth media to facilitate adherence of aspirated cells to the flask surface. After one week, the remaining media/cell suspension was completely removed, and the flask surface was washed with PBS + 1% Pen/Strep several times before refeeding with culture medium. The medium was changed every 2–3 days until 80–90% confluency.

An approximately one-gram sample of periosteal tissue was snap-frozen in liquid nitrogen immediately after collection, then stored at −80 °C for RNA isolation. The remaining periosteum was diced into small pieces, then transferred into 0.2% collagenase (Collagenase type II; Worthington Biochemical Crop., Lakewood, NJ, USA) in growth medium (10 mL per gram of tissue) and digested in a shaking incubator (C24, New Brunswick Scientific, Edison, NJ, USA) at 200 rpm at 37 °C for 4 h. After digestion, the solution was passed through a 40 μm filter (Corning) to remove particulate debris. The filtrate was centrifuged at 300× *g* for 10 min, as above. The cell pellet was resuspended in growth medium, and the cell number was measured with a hematocytometer, using trypan blue (Corning) exclusion to discriminate between viable and dead cells. Cell viability was routinely greater than 95%. Primary cultures were seeded in 100 mm culture dishes at 5 × 10^3^ cells/cm^2^ in growth medium.

### 2.3. Cellular Expansion and Proliferation Assays

When primary bone marrow (BM)- and periosteum (PO)-derived cell monolayers reached 80–90% confluence, the medium was removed, the cell monolayers were rinsed in calcium-free PBS and cells were detached using a brief incubation with 0.05% trypsin/EDTA (Corning). Ten volumes of growth medium were added to deactivate the trypsin/EDTA. The cell suspensions were then transferred to a sterile conical tube and centrifuged at 300× *g* for 10 min to pellet the cells. The cell pellets were resuspended in fresh growth medium, and cell counts were obtained as above. The cells were reseeded at 5 × 10^3^ cells/cm^2^.

Both primary BM and PO cell populations were expanded through two passages to enrich for highly and persistently replicative progenitor cells and to provide sufficient cells for P3 osteogenesis experiments after identical passage conditions. The number of cells isolated from each collection, the numbers seeded and recovered at each passage, and the time required for expansion to confluence were recorded. These values were used to calculate time to confluence and population doubling times (PDTs), expressed as ‘mean days for each population-doubling’ during each passage [25].

### 2.4. In Vitro Osteogenesis

Third passage BM and PO cells were seeded at 10,000 cells/cm^2^ and maintained in growth media until approximately 70% confluence was reached. Control cultures remained in growth media, while osteogenic cultures were transferred into osteogenic media (OM), consisting of growth medium supplemented with 50 μg/mL L-ascorbic acid 2-phosphate magnesium salt hydrate (Sigma-Aldrich, St. Louis, MO, USA), 1 mM β-glycerophospate (Sigma-Aldrich, St. Louis, MO, USA), and 100 nM dexamethasone (Sigma-Aldrich). In subsequent experiments, PO cells in OM were also supplemented with 100 ng/mL recombinant human bone morphogenetic protein 2 (BMP-2; GeminiBio). Cultures were maintained for up to 10 days, during which media was replaced every 2–3 days. Cells were collected for RNA isolation and subsequent reverse transcription-quantitative real-time polymerase chain reaction (RT-qPCR), alkaline phosphatase (ALP) bioactivity assay, and Alizarin Red staining for matrix mineralization at days 5 and 10.

### 2.5. Reverse Transcription and Quantitative PCR

Total RNA was isolated using the phenol-based dissociation agent, TRIzol^®^ (Invitrogen-Thermo Scientific), according to the manufacturer’s recommended protocol. Lysates were homogenized for 30 s using an Ultra-Turrax T25 homogenizer (Janke & Kunkel, IKA Labortechnik, Wilmington, NC, USA) prior to adding chloroform to the reagent. RNA concentrations were quantified using a NanoDrop 2000 spectrophotometer (Thermo Scientific).

One microgram of total RNA was reverse transcribed using the SuperScript^TM^ IV First-Strand Synthesis System kit (Invitrogen-Thermo Scientific). After terminating the reverse transcription reaction, RNA was removed by treating each sample with two units of *E. coli* RNase H and incubating in a 37 °C water bath for 20 min. Final cDNA samples were diluted 1:10 and stored at −20 °C.

Quantitative real-time PCR (qPCR) was performed using 5 μL of the 1:10 diluted cDNA samples and BIO-RAD’s iQ^TM^ SYBR^®^ Green Supermix protocol (BIO-RAD, Hercules, CA, USA). Quantitative PCR reactions were performed using an iCycler iQ^TM^ thermocycler, Optical Module, and Real-Time PCR Detection System Software Version 3.1 (BIO-RAD). Details of the primer pairs are presented in Table 1. All primers were designed for an optimal annealing temperature of 60 °C. This was corroborated by gradient PCR amplification of the targeted amplicons prior to their use for experiments.

Amplicon threshold outcomes were analyzed using the 2^−ΔΔCT^ method [26], adjusted for primer efficiency, and were normalized to expression of the reference gene, Elongation factor 1 alpha (EF1α). For subsequent analyses of specific gene expressions, ‘Control day 5’ expression values in each experiment were assigned a value of ‘1’, and the remaining samples in the experiment were adjusted accordingly.

### 2.6. Alkaline Phosphatase (ALP) Enzymatic Activity Assay

Samples for the ALP bioactivity assay were lysed in 1% Triton X-100 in PBS. The samples were briefly homogenized, as above, left on ice for 30 min, and then centrifuged at 1260 rcf and 4 °C for 15 min (Centrifuge 5415 R, Eppendorf, Enfield, CT, USA). The supernatant was transferred to a new microcentrifuge tube and stored at −20 °C until the assay was performed.

ALP activity was measured using the LabAssay^TM^ ALP kit (Wako Pure Chemical Corporation-Fuji Film, Osaka, Japan) following the manufacturer’s recommended protocol. Experimental sample activities were calibrated against a standard curve, produced by serial dilutions of the Standard Solution (0.5 mmol/L *p*-Nitrophenol). Transparent 96-well microplates (Corning) were used for the assays. The enzymatic reactions were routinely run for 15 min at 37 °C. ALP activities were measured at 405 nm wavelength using a FLUOstar OPTIMA microplate reader and Version 5.10 software (BMG LabTech, Cary, NC, USA).

ALP activity of each sample was normalized to DNA concentration, reflecting cell number in the lysate, by dividing the activity (units/μL/minute) by the DNA concentration (μg/μL). DNA was measured with the Quant-iT^TM^ PicoGreen^®^ dsDNA kit (Invitrogen, Carlsbad, CA, USA), based on the manufacturer’s recommended protocol, using black-sided, clear-bottom 96-well plates (Corning). Fluorescence was measured at 485 nm excitation wavelength using a FLUOstar OPTIMA microplate reader, as above.

### 2.7. Alizarin Red Staining

A 2% Alizarin Red-S solution (Sigma-Aldrich) was prepared in sterile-filtered water and adjusted to a pH of 4.1–4.3. The stain was filter-sterilized to remove any debris and stored at 4 °C when not in use. Cell monolayers were washed in PBS, then fixed in 4% paraformaldehyde for 30 min at room temperature. After fixation, cells were washed three times with PBS before sufficient Alizarin Red-S solution was applied to submerge the monolayers. Plates were incubated for 20 min at room temperature on a plate shaker to distribute stain evenly. The stain was rinsed off using sterile-filtered water and representative images of the stained cells were acquired using a DMIL microscope, PFC320 digital camera, and supporting Leica Application Suite software (LEICA LAS Version 2.6.R1; Leica Microsystems Inc., North Deerfield, IL, USA).

### 2.8. Statistical Analyses

All data were analyzed using GraphPad Prism Version 5.00 (GraphPad Software, INC, Boston, MA, USA). Normally distributed data sets, confirmed by the Kolmogorov–Smirnov tests, were expressed as mean + the standard error of the mean (SEM). The normality of data set distributions was confirmed by the Kolmogorov–Smirnov test. Within each cell type (BM or PO), changes in ‘time to confluence’ and ‘population doubling times’ were analyzed by one-way repeated measures analysis of variance (ANOVA). The effects of time and medium type on osteogenic gene expression and ALP activity in each cell type were analyzed by two-way repeated measures ANOVA. Where indicated, specific changes in relation to ‘Control day 5’ values were determined using Dunnett’s post hoc tests. Paired *t* tests were used to compare specific differences between BM and PO and between periosteal tissue and P2 cell values. Statistical significance was assigned at *p* < 0.05.

## 3. Results

### 3.1. In Vitro Cell Expansion

**Figure 1:**
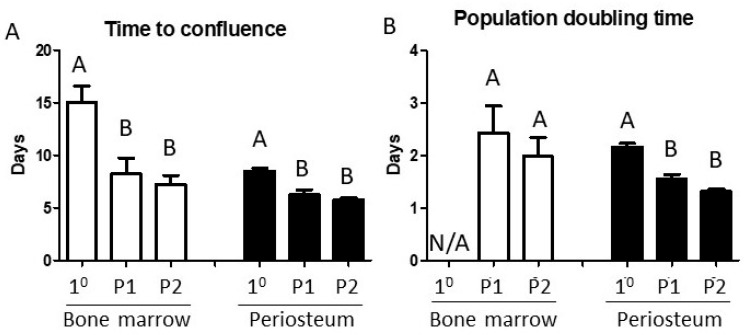
In vitro proliferation characteristics of bone marrow- and periosteum-derived cells during monolayer expansion. (**A**) Time to confluence of primary (1°), passage 1 (P1), and passage 2 (P2) bone marrow (white columns) and periosteal (black columns) cultures. (**B**) Population doubling times of bone marrow- and periosteum-derived cells. PDTs could not be calculated from the primary bone marrow cultures. Repeated measures one-way ANOVAs, mean + SEM, *n* = 5, *p* < 0.05. Within each cell type, groups with similar values are designated by the same letter. N/A, not applicable.

Time to confluence and PDT outcomes are presented in Figure 1. Primary BM aspirates yielded 22.7 +/− 1.08 × 10^6^ nucleated cells/aspirate, although the great majority of these cells are typically of hematopoietic lineage and would not contribute to the adherent cell population that proliferated in primary cultures. Consequently, the time to confluence of primary BM cultures required approximately two weeks, with subsequent passages significantly more rapid (*p* = 0.0014), requiring around half this time. Population doubling times (PDT) could not be calculated from primary BM aspirates but mean P1 and P2 PDTs were 2.43 and 1.98 days, respectively. Primary periosteal cell cultures required around 8 days to reach confluence, reflecting a mean PDT of 2.17 days. Subsequent passages reached confluence more rapidly (*p* = 0.075), with mean PDTs of 1.55 and 1.31 days for P1 and P2 cultures, respectively (*p* = 0.0002).

**Figure 2:**
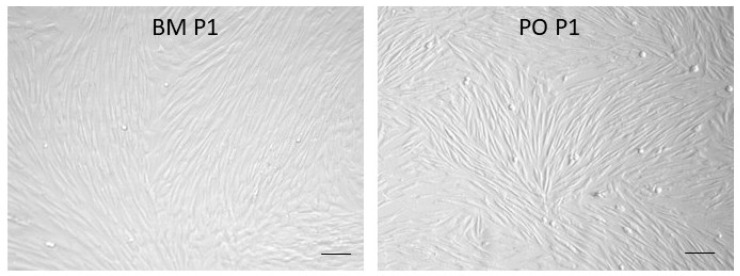
Morphologies of passage 1 (P1) BM and PO cells at confluence. Images were captured using a 10× objective (25× magnification). Size bars = 100 μm.

By P1, the cell morphologies of BM and PO cells were highly similar, and this persisted throughout subsequent passages (Figure 2).

### 3.2. In Vitro Osteogenesis

**Figure 3:**
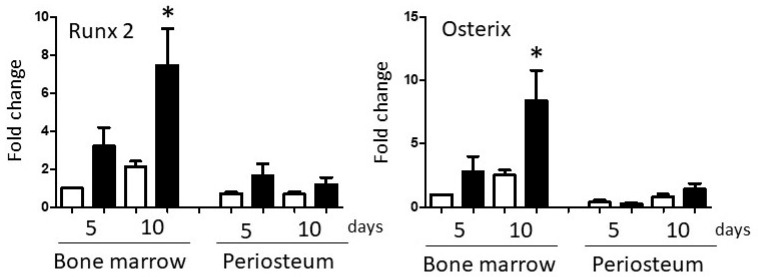
Changes in osteogenic transcription factor expression. Runx2 (left panel) and Osterix (right panel) mRNA expression in P3 bone marrow- and periosteum-derived cells maintained in Control (white columns) or osteogenic (black columns) medium for up to 10 days. In each experiment, BM ‘Control day 5’ threshold cycle values were designated as ‘1’, and other data sets adjusted accordingly. Within each cell type, values significantly different from day 5 Control values are indicated by asterisks. Repeated measures two-way ANOVAs, mean + SEM, *n* = 5, *p* < 0.05.

Basal Runx2 and Osterix mRNA expression in P3 BM and PO cultures were similar (Figure 3); however, the response of BM and PO cells to osteogenic culture conditions differed greatly. BM cells up-regulated expression of both transcription factors, reaching statistical significance by day 10 (Runx2 *p* < 0.001, Osterix *p* < 0.001). In contrast, there was little or no change in expression of either gene in PO cultures at either time-point.

**Figure 4:**
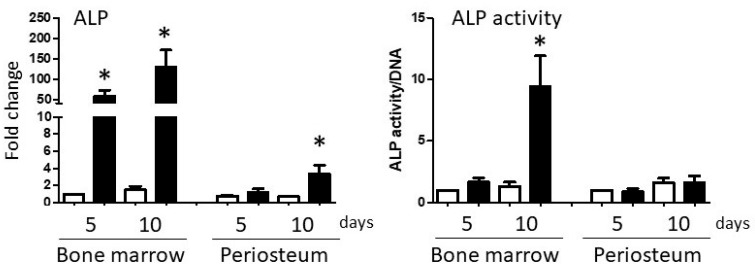
Changes in alkaline phosphatase (ALP) expression and activity. ALP mRNA expression in P3 BM and PO cultures in Control (white columns) and osteogenic (black columns) medium are shown in the left panel. ALP enzymatic activity outcomes are shown in the right panel. To normalize ALP transcriptional and enzyme activity data sets, bone marrow Control day 5 threshold cycle or activity values were designated as ‘1’, and other data sets adjusted accordingly. Within each cell type, values significantly different from day 5 Control values are indicated by asterisks. Repeated measures two-way ANOVAs, mean + SEM, *n* = 5, *p* < 0.05.

Changes in ALP expression and activity in BM and PO cultures were very similar to the osteogenic transcription factor expression profiles above. In BM cultures, ALP mRNA expression was dramatically up-regulated at both time-points (*p* = 0.001), increasing 150-fold by day 10 (Figure 4). Induction of ALP enzymatic activity was less impressive but reached statistical significance on day 10 (*p* = 0.001). In PO cultures, ALP mRNA levels were significantly elevated at day 10 (*p* < 0.01) but increased by only 4-fold over basal expression. PO cell ALP enzymatic activity did not change at either time-point.

**Figure 5:**
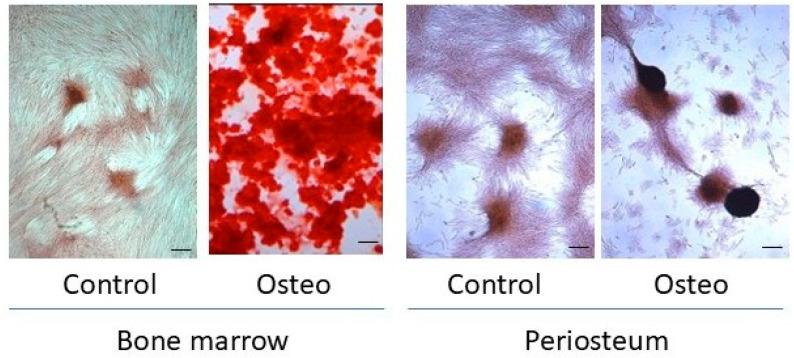
Day 10 matrix characteristics. Matrix mineralization by P3 BM and PO cells maintained in Control or osteogenic (Osteo) medium for 10 days was assessed by Alizarin Red staining. Size bar = 100 μm.

Alizarin Red staining for mineralized matrix secretion corroborated the ALP outcomes. By day 10, BM cultures maintained in osteogenic medium developed dense multicellular aggregates that stained strongly with Alizarin Red, reflecting matrix mineralization (Figure 5). There was some evidence of monolayer contraction and cell aggregation in PO cultures, but Alizarin Red retention was negligible.

#### 3.2.1. Differential Expression of BMP-2 in BM and PO Cell Cultures

**Figure 6:**
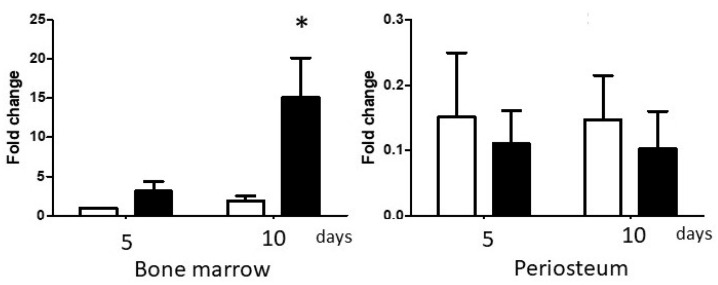
BMP-2 mRNA expression in BM and PO cell cultures. P3 BM and PO cell cultures were maintained in Control (white columns) and osteogenic (black columns) medium for up to 10 days. In each experiment, BM ‘Control day 5’ threshold cycle values were designated as ‘1’, and other values adjusted accordingly. The Y axis scales in the two graphs are consistent in that the mean expression level of BMP-2 in the PO Day 5 Control group was 0.15, relative to BM expression (normalized to 1.0). Within each cell type, values significantly different from day 5 Control values are indicated by asterisks. Repeated measures two-way ANOVAs, mean + SEM, n = 4, *p* < 0.05.

The lack of a robust osteogenic response by PO cells was surprising. Several studies have demonstrated the critical importance of BMP-2 expression and activity in periosteal osteogenesis in both appositional bone growth and fracture repair [27,28,29,30]. Therefore, we examined the expression of BMP-2 in P3 BM and PO cultures. Mean BMP-2 expression in day 5 Control BM cultures was 6-fold higher than in PO cultures. Further, BMP-2 expression was up-regulated in BM osteogenic cultures, reaching statistical significance at day 10 (*p* < 0.01). In contrast, there was no increase in BMP-2 expression in PO cultures (Figure 6). By day 10, the mean BMP-2 expression by BM cells maintained in osteogenic medium was 150 times that of PO cells.

**Figure 7:**
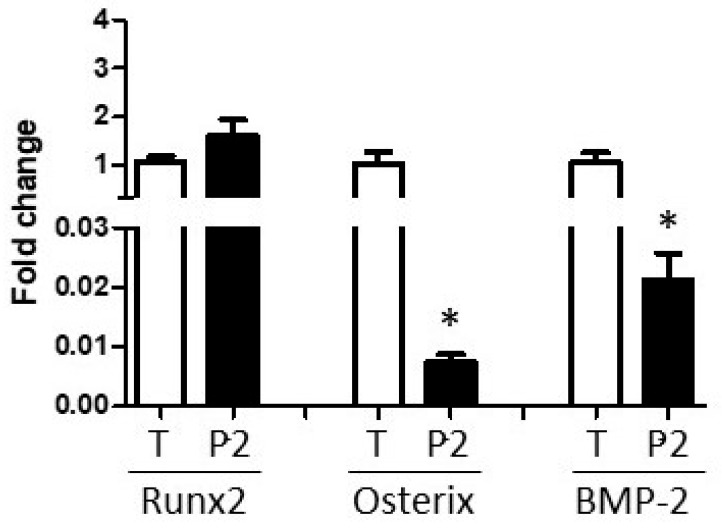
Impact of periosteal cell isolation and expansion on Runx2, Osterix, and BMP-2 expression. The mean tissue (T) sample threshold cycle of each gene was assigned a value of ‘1’ and the donor-paired expression levels at the time of passage 2 (P2) lifting were adjusted accordingly. P2 values significantly different from tissue expression are indicated by asterisks. Paired *t* tests, mean + SEM, *n* = 5, *p* < 0.05.

To address this issue further, the mean ‘in vivo’ expression of Runx2, Osterix, and BMP-2, as measured in the periosteal tissue samples snap-frozen at the time of collection, was compared with the expression of these target transcripts in PO cells at the time of transfer to P3 experimental cultures. Runx2 expression did not change (*p*-value = 0.13), but both Osterix and BMP-2 transcripts were profoundly down-regulated during the cell isolation and expansion process (Figure 7; Osterix *p*-value = 0.014. BMP-2 *p*-value = 0.006). Further, expression levels of these critical osteogenic genes were not restored by transfer to osteogenic culture conditions (Figure 3 and Figure 6).

#### 3.2.2. Exogenous BMP-2 Administration Does Not Restore Periosteal Cell Osteogenic Capacity

**Figure 8:**
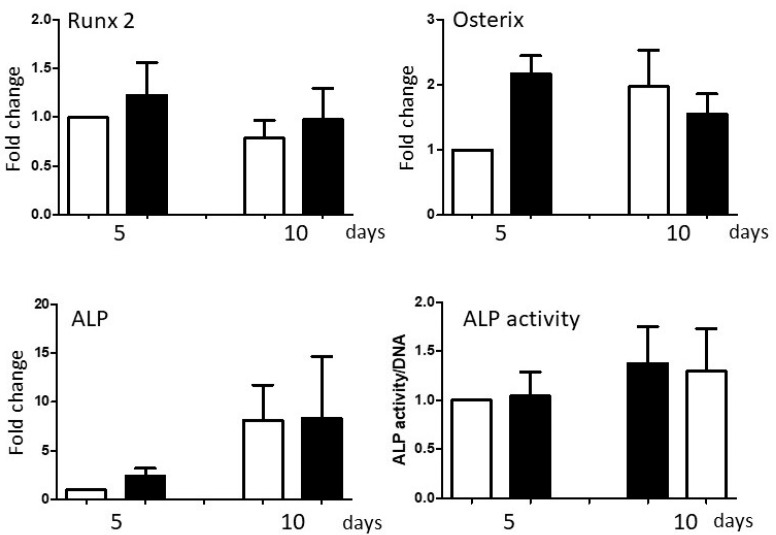
Impact of exogenous BMP-2 administration on periosteal cell osteogenesis. Expression of Runx2, Osterix, and ALP mRNAs and ALP activity were measured in P3 PO cells maintained in osteogenic medium (white columns) or in osteogenic medium supplemented with 100 ng rhBMP-2/mL (black columns) for up to 10 days. To normalize outcomes across individual donor experiments, ‘osteogenic medium day 5’ threshold cycle values or activities were designated as ‘1’, and other data sets adjusted accordingly. Repeated measures two-way ANOVAs, mean + SEM, *n* = 5, *p* < 0.05.

Given the reduced expression of BMP-2 in P3 PO cells, the final series of experiments was carried out to determine whether administration of exogenous recombinant BMP-2 protein can restore PO cell osteogenic capacity. The addition of 100 ng rhBMP-2/mL of osteogenic medium did not alter expression of Runx2, Osterix or ALP mRNAs, or impact the induction of ALP enzymatic activity within the 10-day time frame of the experiments (Figure 8).

**Figure 9:**
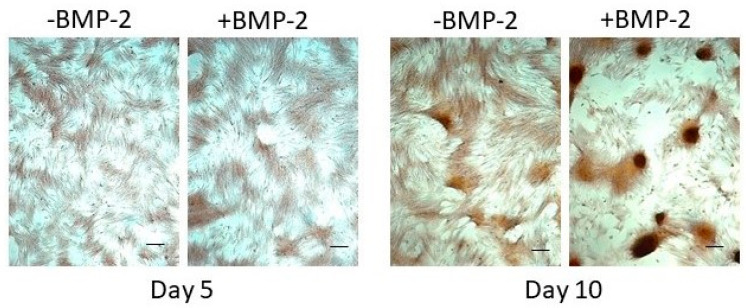
Impact of exogenous BMP-2 administration on matrix mineralization. Matrix mineralization by P3 PO cells maintained in osteogenic medium alone (−BMP-2) or osteogenic medium supplemented with 100 ng rhBMP-2/mL (+BMP-2) for up to 10 days was assessed by Alizarin Red staining. Size bar = 100 μm.

Consistent with the transcriptional and enzymatic activity outcomes, co-administration of rhBMP-2 did not substantively affect matrix mineralization by P3 PO cells, although increased cell aggregation was evident in some experiments (Figure 9).

## 4. Discussion

This study was designed to compare the in vitro osteogenic activities of equine bone marrow- and periosteum-derived cells, two cell populations with robust in vivo osteogenic capacities. The study emphasizes the value of using equine-sourced tissues and cells for comparative studies, since large quantities of tissues and cells can be collected from individual donors to support statistically powerful ‘within individual’ comparisons in a species that is histo-anatomically, biomechanically, and clinically referable to humans [31]. The lower limb anatomy of horses provides easy access to large areas of periosteum, distinct from other potentially contaminating tissues such as skeletal muscle, fascia, and fat. Consistent with our previous findings, BMs exhibited strong indicators of osteogenic differentiation within 10 days of culture [32]. Contrary to our expectations, and despite the findings of Devesa et al. that BM and PO progenitors have very similar molecular signatures, PO cells exhibited negligible osteogenic ability under standard in vitro osteogenic conditions over the same time frame [33]. This result is consistent with those of several published studies and comprehensively disproves our hypothesis [34,35,36,37,38].

The cellular composition of primary bone marrow aspirates is predominantly made up of hematopoietic lineage cells, with mesenchymal progenitor cells present at very low concentrations, hundreds of cells/mL of aspirate, and less than 0.1% of the nucleated cell population [39,40]. BM enrichment from primary aspirates is dependent on the initial attachment of progenitors to cell culture plastic and subsequent rapid and sustained proliferation of these cells. As seen in this and many other studies, sustained in vitro expansion generates a cell population with robust osteogenic capacity, along with several other lineages. Accepting this, there is considerable heterogeneity in BM cell subpopulations with distinct lineage trajectories [41]. Periosteum classically comprises an inner cambium layer containing committed chondro-osteoprogenitors directly adjacent to the cortical bone surface and an outer fibrous layer [42]. This histological distinction may be less relevant from a functional perspective, since cells residing in the outer ‘fibrous’ periosteal layer also have chondro-osteogenic phenotypic capacity with appropriate stimuli [43].

No immuno-phenotypic characterization or selection was carried out on either cell population for this study. It is possible that persistence of ‘non-target’ cells during expansion impacted osteogenic expression of the P3 populations. As mentioned above, multi-passage in vitro expansion of BM aspirates enriches for adherent and persistently proliferative progenitor cells. The primary PO isolates were expanded using the same protocol, but it is possible that PO cell expansion did not exert the same selective enrichment of progenitor cells that occurs in BM cultures. Acknowledging this possibility, the periosteal tissues collected from the cortical bone surfaces with a periosteal elevator expressed comparatively high levels of Runx2, Osterix, and BMP-2 (Figure 7), reflecting inclusion of osteo-progenitors in the tissue samples, and consistent with the findings of Brownlow et al. [44]. Cell viability after PO tissue digestion was routinely greater than 95%, suggesting that a selective loss of PO osteo-progenitors is an unlikely explanation for the study outcome. Despite the initially cellular heterogeneity in both BM aspirates and PO tissues, the monolayer cultures exhibited comparable proliferative indices (Figure 1) and were remarkably homogeneous and cyto-morphologically similar (Figure 2) by P1, consistent with the very similar molecular signatures of these cell populations [33]. The phenotypic consequences of PO cell isolation and in vitro expansion will need to be investigated in more detail to develop protocols that sustain and optimize chondro-/osteo-progenitor enrichment prior to clinical applications.

The termination of the osteogenesis experiments on day 10 was based on the osteogenic behavior of BM cultures. Other published studies investigating periosteal cell osteogenic differentiation have used culture times as long as 30 days, and it is possible that periosteal osteogenesis would have occurred at later time-points if the cultures had been continued [2,31,45,46]. This is supported by the observation that PO cells in osteogenic medium were generating large, multicellular aggregates by day 10, a process that precedes mineralization (Figure 5). Senescence is known to compromise periosteal osteogenic capacity; however, in both cell groups, proliferation indices increased from primary to P2 expansion events, suggesting that senescence was not an overall limitation to cell expansion or subsequent differentiation capacity. Further, the three passages used to expand the experimental cell populations prior to the informative osteogenic differentiation cultures are well below the documented onset of senescence during in vitro monolayer expansion of periosteal cells, and periosteal differentiation capacities have been shown to persist through extensive passages and population doublings in other studies [47,48,49,50].

The process of PO cell isolation and monolayer expansion through three passages dramatically reduced expression of the transcription factor, Osterix, which is mandatorily required for osteogenesis, and also resulted in profound down-regulation of BMP-2 expression, a ligand required for appositional bone growth and fracture repair [28]. Although periosteal cells represent a more lineage-committed cell population along osteogenic and chondrogenic lineages than multi-lineage competent bone marrow progenitors [42], it is clear that cell isolation and in vitro expansion have profoundly different effects on PO and BM cells [1,6]. The fact that exogenous BMP-2 delivery was ineffective in restoring periosteal cell osteogenesis was unexpected, given the importance of BMP-2 for periosteal osteogenesis in developmental and reparative contexts, BMP expression profiles in fracture callus, and clinical use recombinant BMP-2 protein for bone repair [23,25].

Collectively, these findings suggest that BMP-2 activity, although necessary, is not sufficient for in vitro periosteal osteogenesis. Other growth factors, receptors, and signaling intermediates necessary to activate osteogenic differentiation, such as other TGF-β superfamily and Wnt family members, biomechanical and physical cues, are likely also impacted by the cell isolation and in vitro expansion process. There is evidence from in vivo studies that periosteal tissue explants retain osteogenic activity to a far greater degree than isolated PO cell preparations, suggesting that osteogenic stimuli and physical cues exist within PO tissue and/or the peri-osseous microenvironment that are not sustained during cell isolation [51,52,53,54]. Clinical strategies for periosteal cell-based bone repair will first require that these additional stimuli be identified and techniques developed to retain or restore their activities during isolation and expansion. Based on the outcomes of this study, bone marrow-derived stem cells remain as a superior resource for cell-based approaches to bone repair.

## 5. Conclusions

Bone marrow-derived stem cells exhibited far greater osteogenic capacity than cells isolated from periosteum under standard in vitro osteogenic culture conditions. Cell isolation from periosteal tissue and subsequent monolayer expansion resulted in dramatic down-regulation of Osterix and BMP-2 expression in periosteal cultures. This was not reversed by exposure to osteogenic medium or exogenous BMP-2 supplementation. These outcomes need to be factored into any strategy focused on utilizing periosteal cells for cell-based bone healing applications. Ongoing research should be focused on identifying the factors and contexts that activate and sustain periosteal bone formation.

## Figures and Tables

**Table 1:** Primer pairs sequences used to amplify target cDNAs.

Gene Target	Sense (S) and Anti-Sense (A) Primer Sequences	Amplicon Size
EF1α	S: 5′-CCCGGACACAGAGACTTCAT-3′ A: 5′-AGCATGTTGTCACCATTCCA-3′	328
Runx2	S: 5′-CAGACCAGCAGCACTCCATA-3′ A: 5′-CAGCGTCAACACCATCATTC-3′	177
Osterix	S: 5′-GGCTATGCCAATGACTACCC-3′ A: 5′-GGTGAGATGCCTGCATGGA-3′	207
ALP	S: 5′-TGGGGTGAAGGCTAATGAGG-3′ A: 5′-GGCATCTCGTTGTCCGAGTA-3′	221
BMP-2	S: 5′-TAACCACGCCATTGTTCAGA-3′ A: 5′-ACAACCCTCCACAACCATGT-3′	160

## Data Availability

Data supporting reported results is stored in the corresponding author’s laboratory. The data has not been submitted to any publicly archived data sets. The samples and data sets are currently being used by the Stewart lab in ongoing studies of periosteal biology.

## Abbreviations

The following abbreviations are used in this manuscript:

BM	Bone marrow-derived
IACUC	Institutional Animal Care and Use Committee
PO	Periosteal-derived
ALP	Alkaline phosphatase
ANOVA	Analysis of variance
CM	Centimeter
ML	Milliliter
DMEM	Dulbecco’s Modified Eagle’s Medium
FBS	Fetal bovine serum
Pen/Strep	Penicillin/Streptomycin
PBS	Phosphate-buffered saline
SEM	Standard error of the mean
RCF	Relative centrifugal field
IU	International unit
qPCR	Quantitative real-time polymerase chain reaction
TGF-β	Transforming growth factor beta
